# Equiaxial Strain Modulates Adipose-derived Stem Cell Differentiation within 3D Biphasic Scaffolds towards Annulus Fibrosus

**DOI:** 10.1038/s41598-017-13240-3

**Published:** 2017-10-09

**Authors:** Mostafa Elsaadany, Kayla Winters, Sarah Adams, Alexander Stasuk, Halim Ayan, Eda Yildirim-Ayan

**Affiliations:** Department of Bioengineering, University of Toledo, Toledo, OH USA

## Abstract

Recurrence of intervertebral disc (IVD) herniation is the most important factor leading to chronic low back pain and subsequent disability after discectomy. Efficacious annulus fibrosus (AF) repair strategy that delivers cells and biologics to IVD injury site is needed to limit the progression of disc degeneration and promote disc self-regeneration capacities after discectomy procedures. In this study, a biphasic mechanically-conditioned scaffold encapsulated with human adipose-derived stem cells (ASCs) is studied as a potential treatment strategy for AF defects. Equiaxial strains and frequencies were applied to ASCs-encapsulated scaffolds to identify the optimal loading modality to induce AF differentiation. Equiaxial loading resulted in 2–4 folds increase in secretion of extracellular matrix proteins and the reorganization of the matrix fibers and elongations of the cells along the load direction. Further, the equiaxial load induced region-specific differentiation of ASCs within the inner and outer regions of the biphasic scaffolds. Gene expression of AF markers was upregulated with 5–30 folds within the equiaxially loaded biphasic scaffolds compared to unstrained samples. The results suggest that there is a specific value of equiaxial strain favorable to differentiate ASCs towards AF lineage and that ASCs-embedded biphasic scaffold can potentially be utilized to repair the AF defects.

## Introduction

Intervertebral disc (IVD) degeneration affects 97% of individuals, who are 50 years or older^1^. It causes disc herniation and subsequently compression of the spinal nerves and the adjacent vertebrae^2^. Discectomy is the most common surgical procedure to treat herniation and chronic low back pain^3,4^. Yet, the unrepaired annulus fibrosus (AF) defects following the discectomy lead to accelerated IVD degeneration because of increased inflammation and altered biomechanics^5–8^. Thus, discectomy procedures have up to 27% risk of re-herniation requiring repeated surgeries^3^. The outcome of discectomy can be improved through sealing the AF defects and delivering biologics to the injury site^3,5^. In fact, cell-based therapies are required for AF repair due to the avascular and hypocellular nature of the tissue^9,10^. There is a great demand for a scaffold- and cell-guided AF repair system considering the biological and mechanical requirements for AF tissue while providing structural support.

Promising results were obtained by incorporating bone-marrow derived mesenchymal stem cells (BMSCs) within tissue-engineered scaffolds for AF regeneration^11–14^. However, using BMSCs was associated with potential inflammatory reactions and ectopic bone formation^15,16^ that might be unfavorable for regenerating annulus fibrosus. Therefore, ASCs utilization in regenerating AF has emerged since they overcome the limitations of BMSCs^17–19^. Specifically, ASCs is a very promising cell type of targeting IVD regeneration^20,21^.

Several research groups have focused on conditioning musculoskeletal tissue scaffolds using mechanical loading platforms^17,22,23^ including mechanical strain applications to cell-embedded three-dimensional (3D) scaffolds to regenerate and repair the AF tissue^3,24–30^. Studies showed that physiological mechanical strains stimulated anabolic effects on AF cells *in vitro* while pathological strains resulted in a deleterious response^27^. However, the nature of mechanical cue is very specific to the tissue of interest. Our literature review revealed that even though there are studies linking uniaxial and biaxial strains as well as other mechanical cues to ASCs differentiation^24,31^, there are almost no studies that investigated the effect of equiaxial strain on the differentiation potential of these cells in 3D culture conditions.

In this study, a comprehensive methodology was followed to assess the solo effect of different equiaxial mechanical strains on the matrix organization and the targeted differentiation of ASCs encapsulated within the 3D collagen and biphasic nanofibrous AF scaffold. First, a custom-made equiaxial loading platform (EQUicycler)^32,33^ was employed to apply cyclic equiaxial mechanical strain within the physiological range of the annulus fibrosus tissue^27,28,34–36^. The loaded constructs as well as control (unstrained) groups were evaluated in terms of gene expression, cells morphology, and matrix reorganization. Also, the secreted soluble ECM proteins including sulfated Glycosaminoglycans (sGAG) and collagens were quantified. The obtained data from these assays were used to identify the equiaxial mechanical strain and frequency required to differentiate ASCs towards AF lineage. Upon identifying the most appropriate equiaxial loading modality, the ASCs embedded-biphasic scaffolds were subjected to the selected equiaxial strain and frequency and characterized for cells proliferation, cells morphology, matrix reorganization. Further, region-specific differentiation of ASCs within the biphasic scaffold to the inner and outer regions associated with the native AF tissue was investigated through gene expression analyses. The outcomes of the current study suggest that a) there is a specific value of equiaxial strain and frequency favorable differentiate ASCs towards AF lineage, and b) the ASCs-embedded collagen-PNCOL biphasic scaffold can potentially be utilized to repair the annular defects of the IVD.

## Results

### Effect of equiaxial mechanical strain and frequency on lineage commitment of ACSs within collagen scaffold

**Figure 1:**
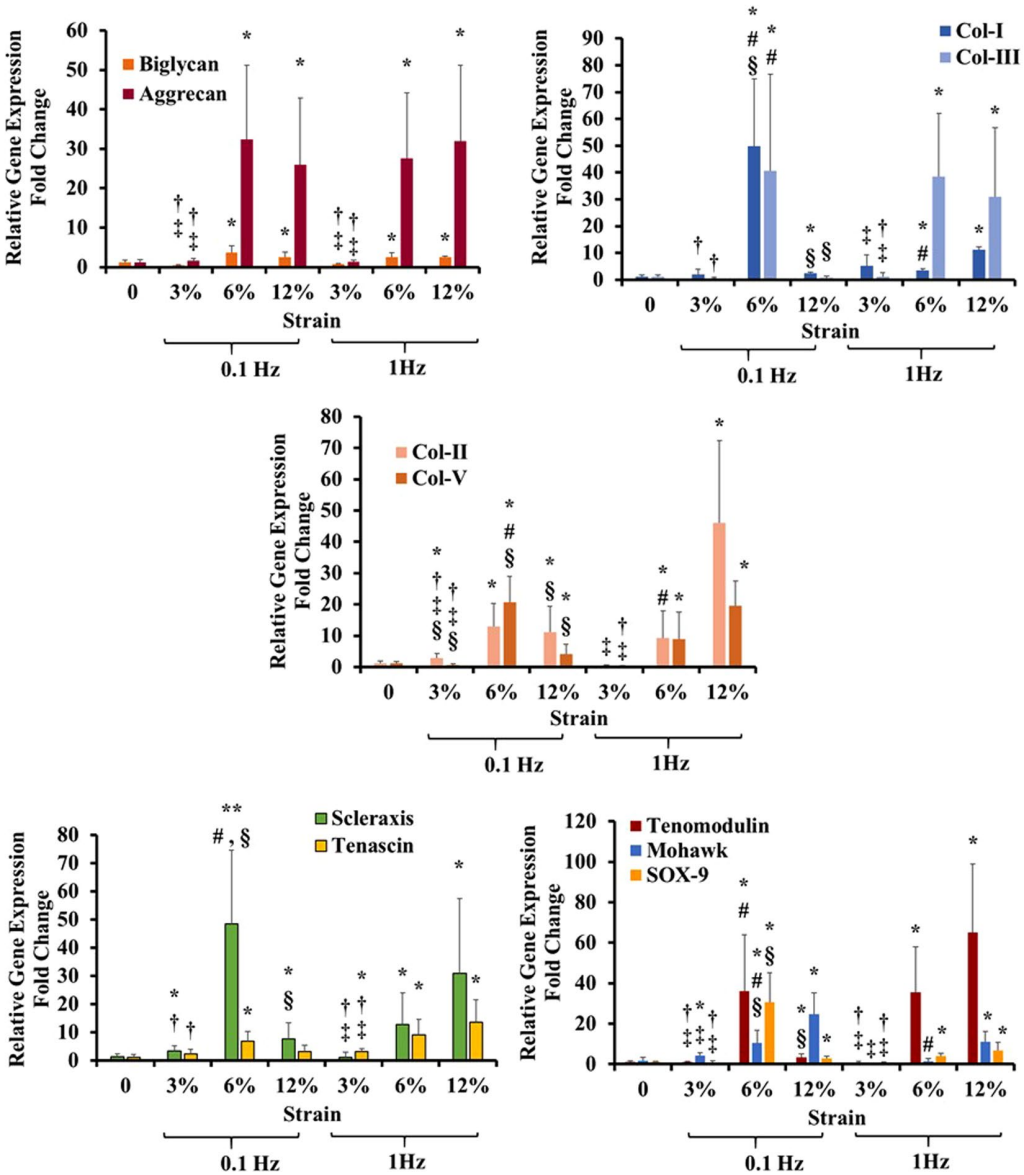
Effect of varying magnitudes and frequencies of equiaxial strain on the gene expression profiles of the ECM proteins and AF tissue markers. The gene expression profiles for the ECM proteins as well as AF markers were upregulated by equiaxial mechanical loading. Data represent the mean fold change (n = 8), and the error bars represents the standard deviation. *Indicated significant difference with respect to 0% strain group (control) with p < 0.05. ^§^Represented significant difference between 0.1 Hz and 1 Hz (at the same strain magnitude), ^†^Represented significant difference between 3% and 6% strain groups (at the same frequency), ^‡^represented significant difference between 3% and 12% strain groups (at the same frequency) while ^#^represented significant difference between 6% and 12% strain groups (at the same frequency), each with p < 0.05.

The effect of varying magnitudes and frequencies of equiaxial strain on the relative expression of ECM proteins was quantified using RT-qPCR. The gene expression profiles were investigated after 7 days of mechanical stimulation at 0% (control), 3%, 6%, or 12% strains at 0.1 Hz or 1 Hz frequencies. Figure 1 shows the relative gene expression fold change of the ECM markers (Aggrecan, Biglycan, Collagen I, II, III, and V) for the loaded and control groups. The results showed that the 6% and 12% strain groups had upregulated ECM markers gene expression compared to control. As for the tenogenic and chondrogenic genes (Scleraxis, Tenascin, Tenomodulin, Mohawk, and SOX-9) that were shown to be expressed in the AF tissue^8,37–39^, the results also demonstrated that the 6% and 12% strain groups had upregulated AF-markers gene expression compared to unstrained samples.

**Figure 2:**
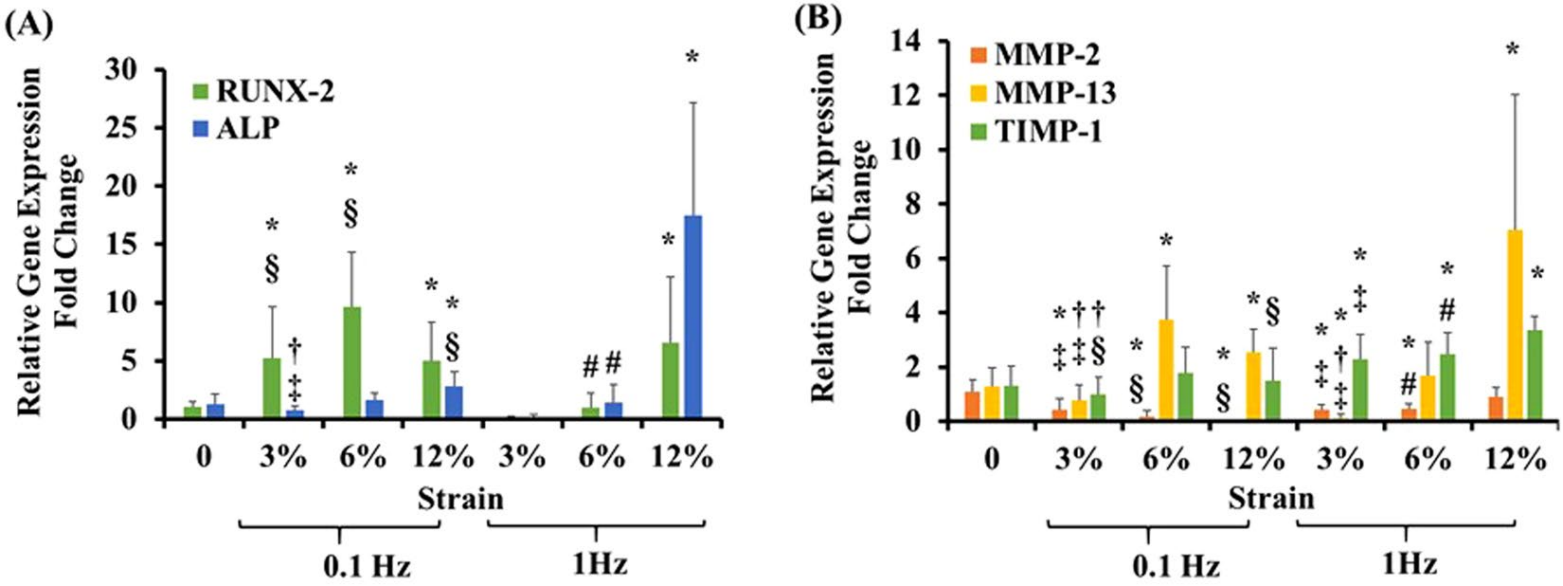
(**A**) Effect of varying magnitudes and frequencies of equiaxial strain on the osteogenic markers expression profiles. Loaded samples showed mixed osteogenic response with 3% and 6% groups loaded at 1 Hz showing no osteogenic expression. Data represent the mean fold change (n = 8), and the error bars represents the standard deviation. *Indicated significant difference with respect to 0% strain group (control). (**B**) Effect of varying magnitudes and frequencies of equiaxial strain on the gene expression profiles of catabolic proteins (MMP-2 and MMP-13) and TIMP-1 that are associated with the AF tissue. Loaded samples showed elevated anabolic response compared to control. *Indicates p < 0.05. ^§^Represented significant difference between 0.1 Hz and 1 Hz (at the same strain magnitude), ^†^represented significant difference between 3% and 6% strain groups (at the same frequency), ^‡^represented significant difference between 3% and 12% strain groups (at the same frequency) while ^#^represented significant difference between 6% and 12% strain groups (at the same frequency), each with p < 0.05.

To investigate the potential osteogenic differentiation of ASCs, as well as the potential catabolic or anabolic activities with varied equiaxial mechanical loading modalities, osteogenic markers (RUNX-2 and ALP), catabolic markers (MMP-2, MMP-13), and tissue inhibitor of metalloproteinase-1 (TIMP-1) genes expression profiles, were examined. Figure 2(A) shows the relative gene expression fold change of RUNX-2 and ALP. For RUNX-2, significantly higher gene expression was observed for all the 0.1 Hz groups. For the 1 Hz groups, only the 12% strain had higher gene expression compared to control. For ALP, only the 12% strain groups were significantly higher than control for both the 0.1 and 1 Hz frequencies. Figure 2(B) demonstrates the effect of different equiaxial loading modalities on the gene expression profiles of catabolic (MMP-2 and MMP-13) and tissue inhibitor of metalloproteinase-1 (TIMP-1) that were associated with AF tissue^35,40^. For MMP-2, significantly lower gene expression was found for all the loaded groups except for the 12% strain at 1 Hz. For MMP-13, we observed higher gene expression profiles for the 12% strain groups loaded at both the 0.1 and 1 Hz frequencies as well as the 6% group loaded at 0.1 Hz. For the low strain groups (3%), the gene expression was significantly lower in the 1 Hz group and was not significant for the 0.1 Hz group.

### Effect of equiaxial mechanical strain and frequency on secretion of ECM proteins from ACSs within collagen scaffold

**Figure 3:**
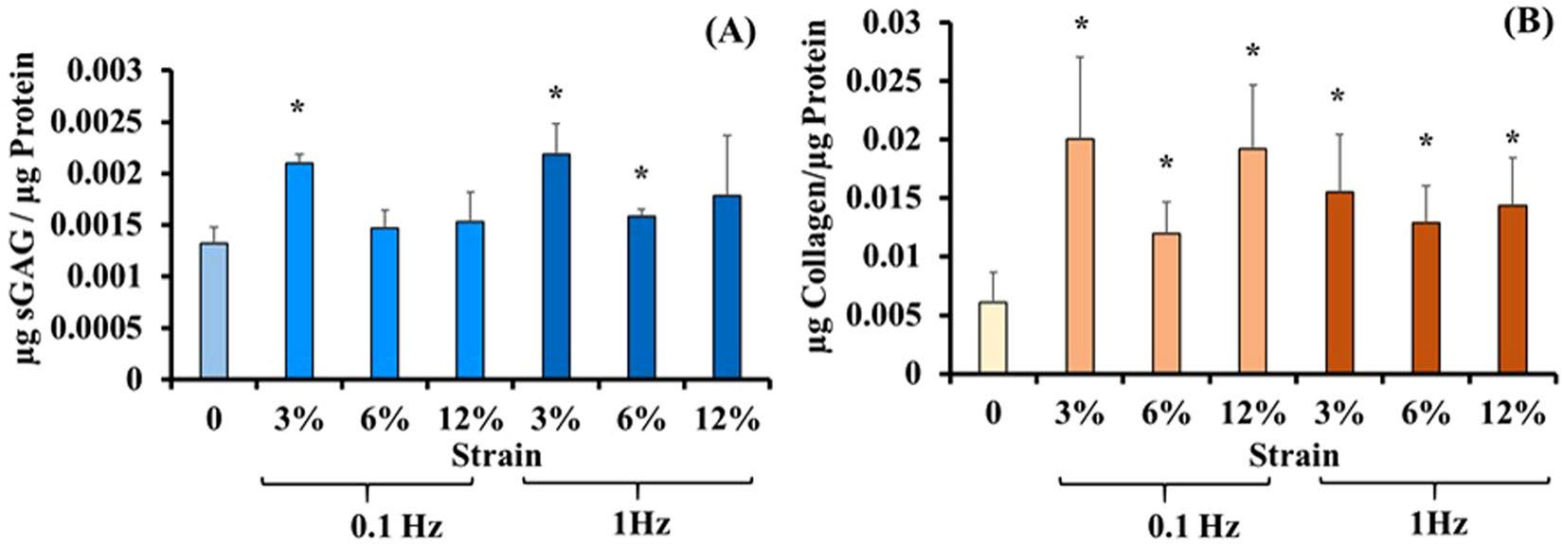
Effect of varying magnitudes and frequencies of equiaxial strain on the amount of soluble secreted EMC proteins in the culture media. (**A**) Normalized secreted sGAG in the cell culture media. Samples loaded at 3% (0.1 Hz) and samples loaded with 3%, 6% at 1 Hz showed higher normalized soluble GAG content in the culture media (**B**) Normalized soluble collagens in the cell culture media. Loaded samples showed higher amounts of soluble collagens in the culture media. Data represents the average (n = 8), and the error bars represents the standard deviation. *Represents statistical difference from the control (unstrained group) with p < 0.05.

The ECM gene expression profiles results were further confirmed by investigating the levels of ECM proteins secretion. The effect of varying magnitudes and frequencies of equiaxial strain on the secreted ECM proteins (sGAG and collagens) was investigated. The results are shown in Fig. 3. We have seen significantly higher normalized sGAG for the 3% (0.1 Hz) as well as for both the 3% and 6% (1 Hz). Furthermore, we noticed significantly higher (~2 to 4 folds) soluble collagens for all mechanically loaded groups compared to control.

### Identifying the optimum mechanical strain magnitude and loading frequency to induce ASCs differentiation towards AF lineage

The results obtained from the gene expression and ECM protein secretion analyses were used to optimize the equiaxial loading conditions. Based on these assays, we concluded that equiaxial loading significantly increased the gene expression of the major AF tissue markers. Also, several loading modalities resulted in higher secreted ECM proteins. Specifically, the scaffold loaded with 6% strain and 1 Hz frequency showed a good level of gene expression for AF tissue markers and some ECM proteins, while showed significantly lower expression of osteogenic differentiation markers (ALP and RUNX-2) and the catabolic proteins genes (MMP-2 and MMP-13) that are associated with AF degeneration. We also observed higher expression of TIMP-1 that was shown to suppress the osteogenic differentiation of mesenchymal stem cells^41^. At the protein level, the 6% strain group loaded at 1 Hz showed higher sGAG and soluble collagens amounts in the scaffolds culture media. Therefore, we concluded that the 6% strain and 1 Hz frequency is the most appropriate loading modality for a potential AF repair strategy and then conducted the histological analysis for the collagen scaffolds and the biochemical assays as well as the histological analysis for the biphasic scaffolds for 6% strain and 1 Hz frequency loading with the control groups kept in no-loading conditions.

### Effect of equiaxial mechanical loading on matrix reorganization and ASCs morphology of ACSs within the collagen scaffold

**Figure 4:**
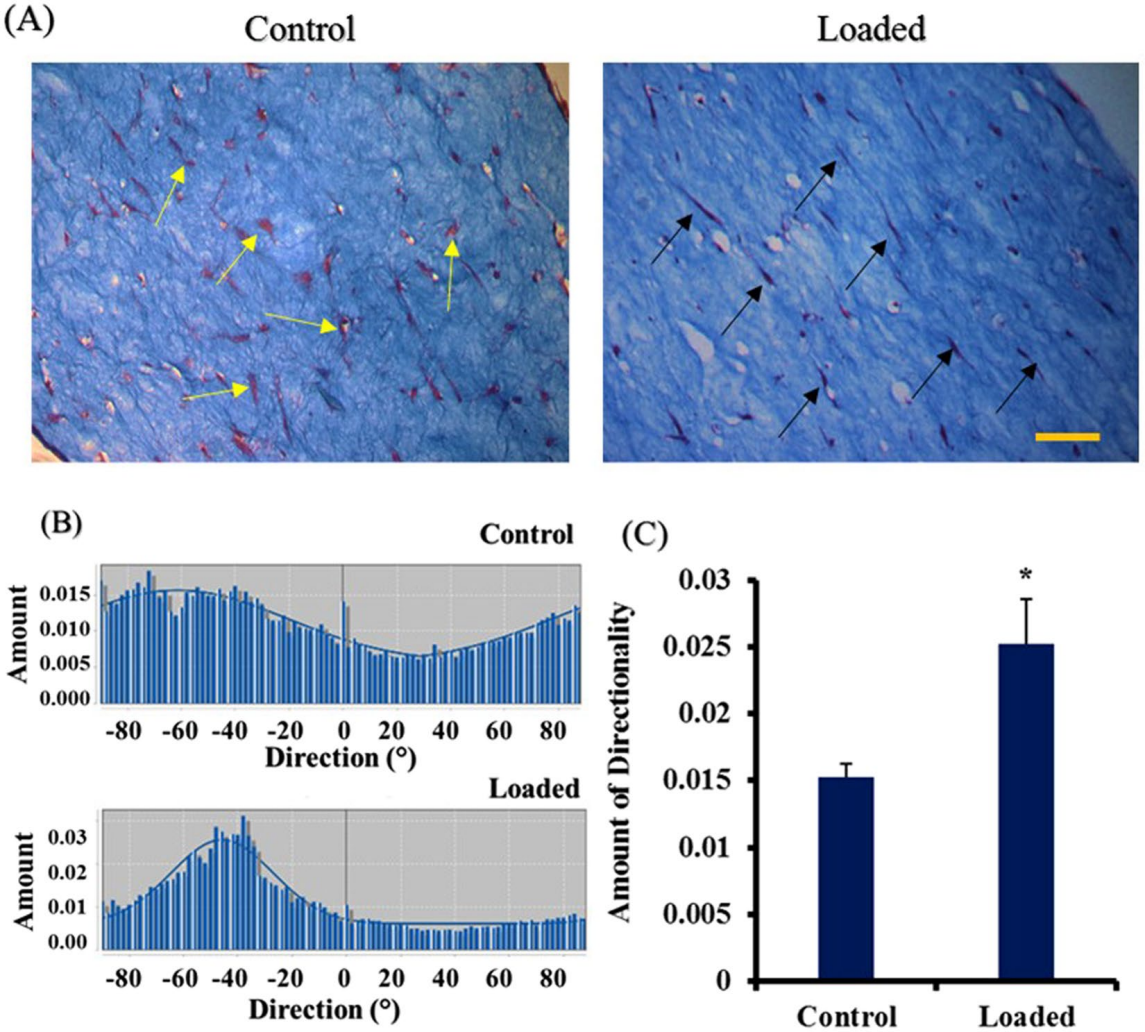
Effect of equiaxial strain on matrix organization and the cells morphology of ASC-encapsulated 3D collagen scaffolds. (**A**) Histological visualization using Mason’s Trichrome staining. Mason’s trichrome stains the collagen fibers with blue color, the cytoplasm of the cell is stained with red, and the nucleus is stained with purple color. Loaded ASCs-encapsulated collagen (6% strain and 1 Hz frequency) showed more aligned fibers and elongated cells compared to control. Scale bar represents 100 µm. Black arrows point to the aligned and elongated cells while yellow arrows point to the round and randomly distributed cells (**B**) Representative directionality histograms for the loaded and control samples. (**C**) The average amount of directionality obtained from various field views (n = 4) through imageJ. Loaded samples showed higher directionality compared to control. *Represents statistical difference (p < 0.05) from the control group. Error bars represent the standard deviation.

To investigate the effect of equiaxial mechanical loading on the matrix reorganization and ASCs morphology within the 3D collagen scaffolds, histological analyses were conducted. Figure 4(A) shows the differences in collagen fibrillar orientation and the morphology of the residing ASCs after seven days of applying 6% strain at 1 Hz loading compared to control (unstrained). We did not observe any physical damage or breakage within the matrix in response to the loading with EQUicycler. The equiaxially loaded collagen construct showed a high degree of aligned fibers, and the cells were aligned along the principal loading direction and demonstrated elongated morphology. On the other hand, the control group (unstrained) showed a higher number of cells and matrix compaction compared to the loaded group. However, the cells had a random distribution within the collagen construct and relatively round morphology. To quantify the degree of the matrix organization, directionality analysis was performed to at least eight field views using ImageJ software (NIH, USA). Figure 4(B) shows representative histograms that represent the degree of orientation of the collagen fibers as indicated by the peak value and the scatter. The directionality histogram of loaded sample showed a distinct peak of higher magnitude, and less scatter compared to the control sample that did not demonstrate a distinctive peak. The amount of directionality is represented in Fig. 4(C). The amount of directionality was significantly higher for the loaded samples compared to control groups with an almost two-folds difference (P < 0.05).

### Effect of equiaxial mechanical loading on secretion of ECM proteins from ACSs within the biphasic scaffold

We have confirmed that 6% strain at 1 Hz equiaxial loading modality promote ASCs AF differentiation, ECM markers expression, and matrix directionality while suppressing osteogenic and catabolic markers. We utilized this fundamental knowledge to create a biphasic mechanically conditioned scaffold that comprised an inner region of ASCs-encapsulated collagen and an outer region of ASCs-encapsulated PNCOL for AF tissue regeneration.

**Figure 5:**
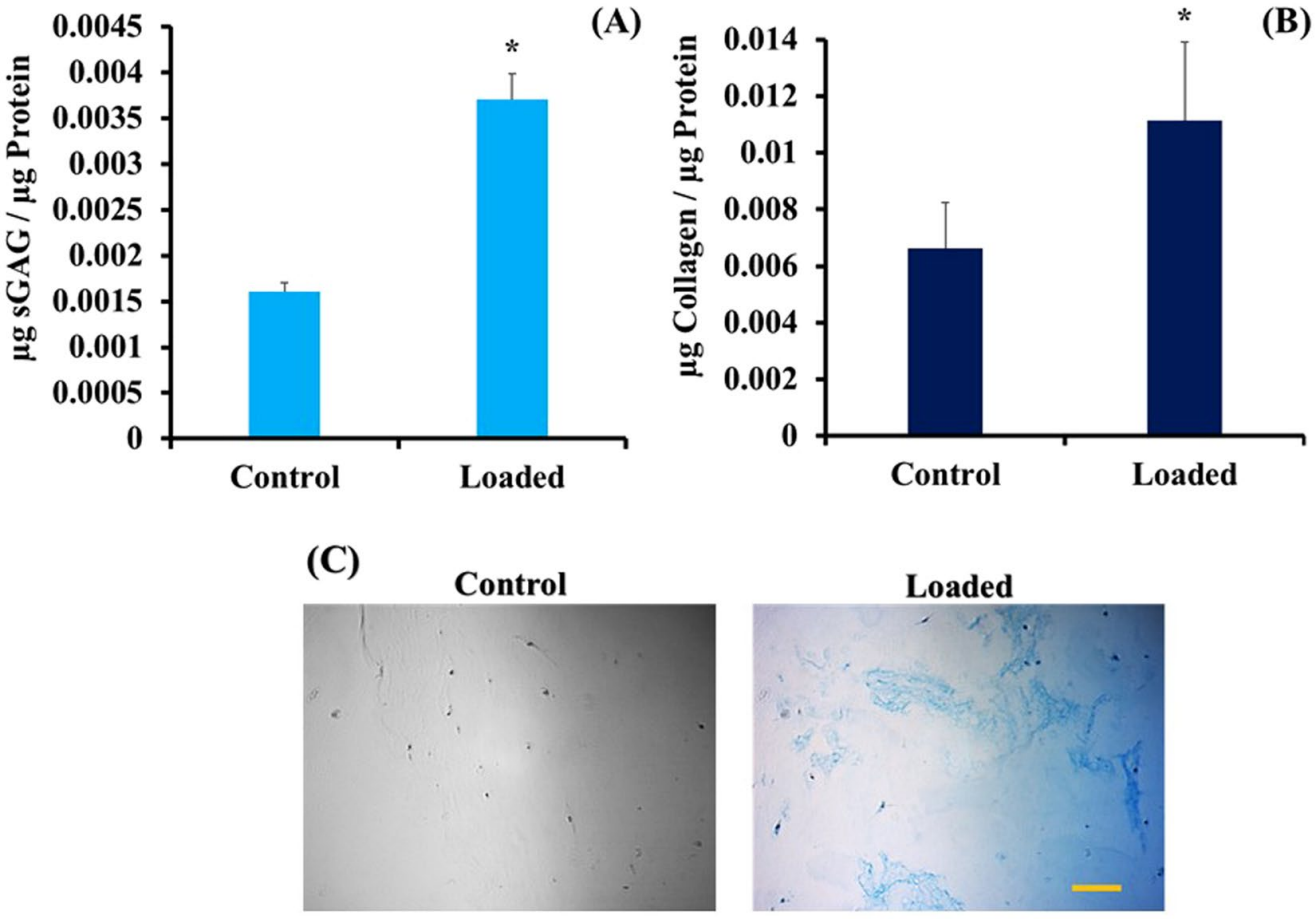
Effect of equiaxial strain on the amount of soluble secreted sGAG (**A**) and Collagens (**B**) in the culture media. Loaded ASCs-collagen-PNCOL biphasic scaffolds showed higher secreted ECM proteins amounts in the culture media. Data represents the average (n = 8), and the error bars represents the standard deviation. *Represents statistical difference from the control (unstrained group) with P < 0.05. (**C**) Histological visualization of the secreted GAG within the biphasic scaffolds using Alcian Blue staining. Alcian Blue dye stains GAGs with blue color. Mechanically loaded samples showed higher amounts of secreted GAG within the scaffolds. Scale bar represents 100 µm.

We first investigated the effect of equiaxial strain on the secreted ECM proteins for soluble sGAG and Collagens using colorimetric assays and GAG content within the scaffold using histology data. The results presented in Fig. 5A show almost four folds increase in the normalized amount of secreted sGAG of the loaded samples (6%, 1 Hz) compared to control (unstrained). In Fig. 5B, there is a two-folds increase in the normalized amounts of soluble collagens of the loaded group compared to control. To confirm the presence of secreted GAG within the scaffolds, histology slides were stained with Alcian Blue (Fig. 5C). There is a difference between the loaded group (6%, 1 Hz) that showed higher GAG secretions compared to control groups that showed little GAG secretions. Alcian Blue staining, as well as DMMB and Sircol assay results, confirmed that mechanical stimulation could induce secretion of ECM at the protein levels within the scaffolds.

### Effect of equiaxial mechanical loading on matrix reorganization and cells morphology of ACSs within the biphasic scaffold

**Figure 6:**
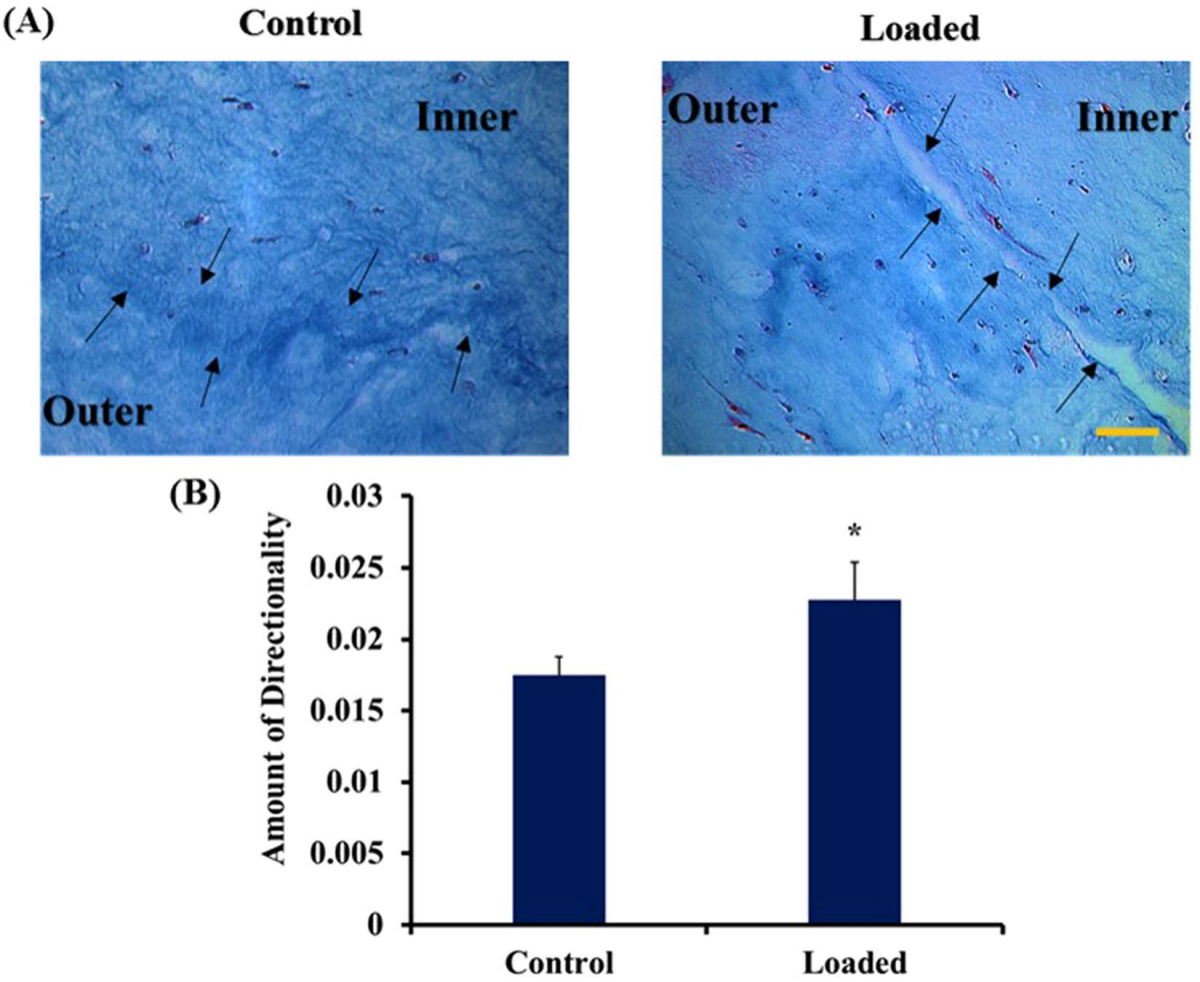
Effect of equiaxial strain on matrix organization and the cells morphology of ASC-encapsulated collagen-PNCOL biphasic scaffolds. (**A**) Histological visualization using Mason’s Trichrome staining demonstrated that loaded samples had higher aligned fibers and elongated cells compared to control samples. The black arrows point to the interface between collagen (inner) and PNCOL (outer) regions of the biphasic scaffold. Scale bar represents 100 µm. (**B**) The average amount of directionality obtained from various field views (n = 4) through imageJ. Loaded samples showed higher directionality compared to control. *Represents statistical difference (p < 0.05) from the control group. Error bars represent the standard deviation.

To investigate the effect of equiaxial loading on the matrix organization and cells morphology of ASCs-encapsulated 3D collagen-PNCOL scaffolds, histological analysis was conducted. Figure 6 demonstrates that the equiaxially loaded biphasic constructs had highly aligned fibers in the collagen portion and less aligned fibers in the PNCOL portion, but the cells were aligned along the principal loading direction and demonstrated elongated morphology in both the inner and outer regions of the scaffolds. Like the collagen scaffolds (Fig. 4), the control group cells showed a random distribution and relatively round morphology within the both the collagen (inner) and PNCOL (outer) regions of the biphasic construct. Also, the control group showed more matrix compaction compared to the loaded group possibly because the higher number of cells that acts to pull the collagen fibers together. The directionality analysis of various fields of view at the interface between collagen and PNCOL (Fig. 6B) shows that the strained samples had higher alignment compared to control group.

### Effect of equiaxial mechanical loading on region-specific gene expression of ACSs within biphasic scaffold

**Figure 7:**
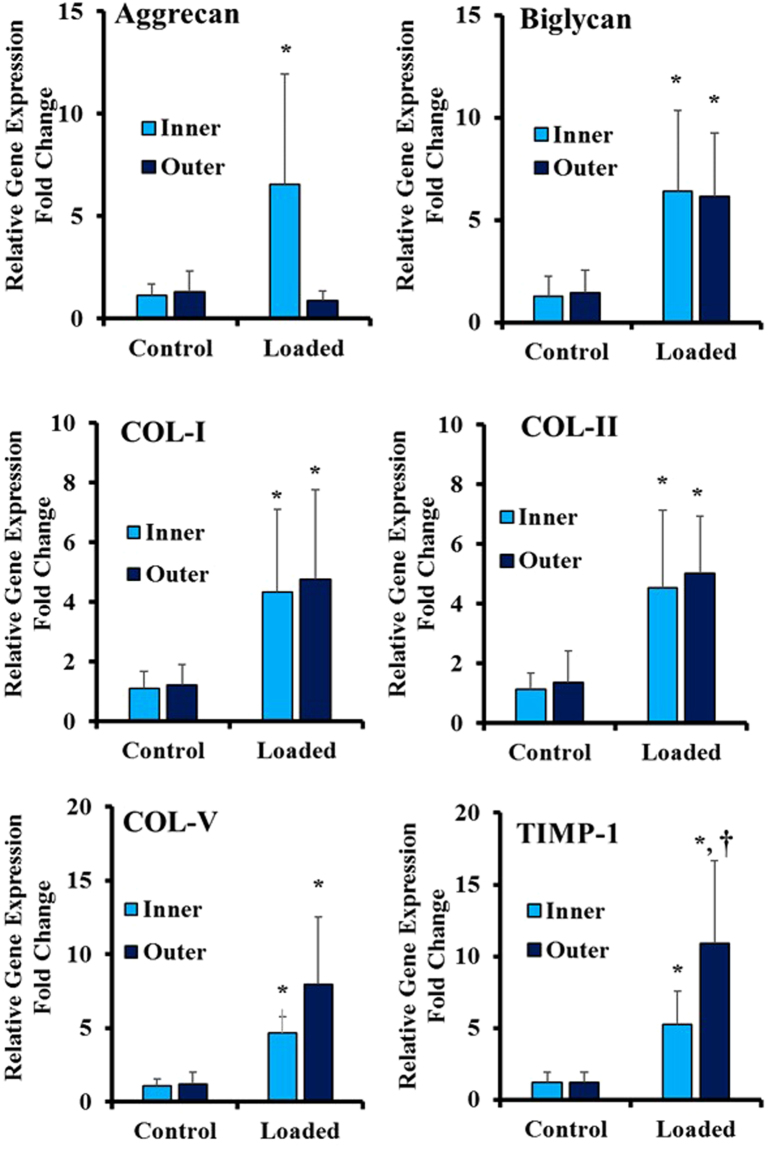
Effect of equiaxial strain on the gene expression profiles of the native AF ECM marker proteins. Loaded samples exhibited higher gene expression compared to control group. Data represent the mean fold change (n = 8), and the error bars represents the standard deviation. *Represents statistical difference from the control (unstrained group) with 0.05 P-value. ^†^Represented a significant difference between the inner and outer regions with a 0.05 P-value.

We further investigated the spatial differentiation of ASCc within the inner and outer regions of the biphasic scaffolds by analyzing the relative gene expression of the AF tissue associated genes by RT-qPCR. Figure 7 shows the AF ECM-associated genes expression fold change for the loaded samples (6%, 1 Hz) compared to control (unstrained) groups. Significantly, for the large proteoglycan (Aggrecan), we found higher gene expression only in the collagen portion of the biphasic scaffold compared to control. As for the smaller proteoglycan (Biglycan), there was higher gene expression in both Collagen and PCNOL regions. Collagen Type-I and Type-II genes had higher gene expression in both regions of the biphasic scaffold compared to control. For Collagen Type-V, higher gene expression was observed for the inner region and the outer region compared to unstrained samples. No statistical difference between the inner and outer regions in the expression of collagens. Finally, we investigated the expression of the tissue inhibitor of metalloproteinase-1 (TIMP-1) since it is associated with the anabolic activities in the AF tissue as well as suppression of osteogenic differentiation of mesenchymal stem cells^41^. For TIMP-1, we found significantly higher expression in the inner and outer regions of the scaffold compared to control, respectively.

**Figure 8:**
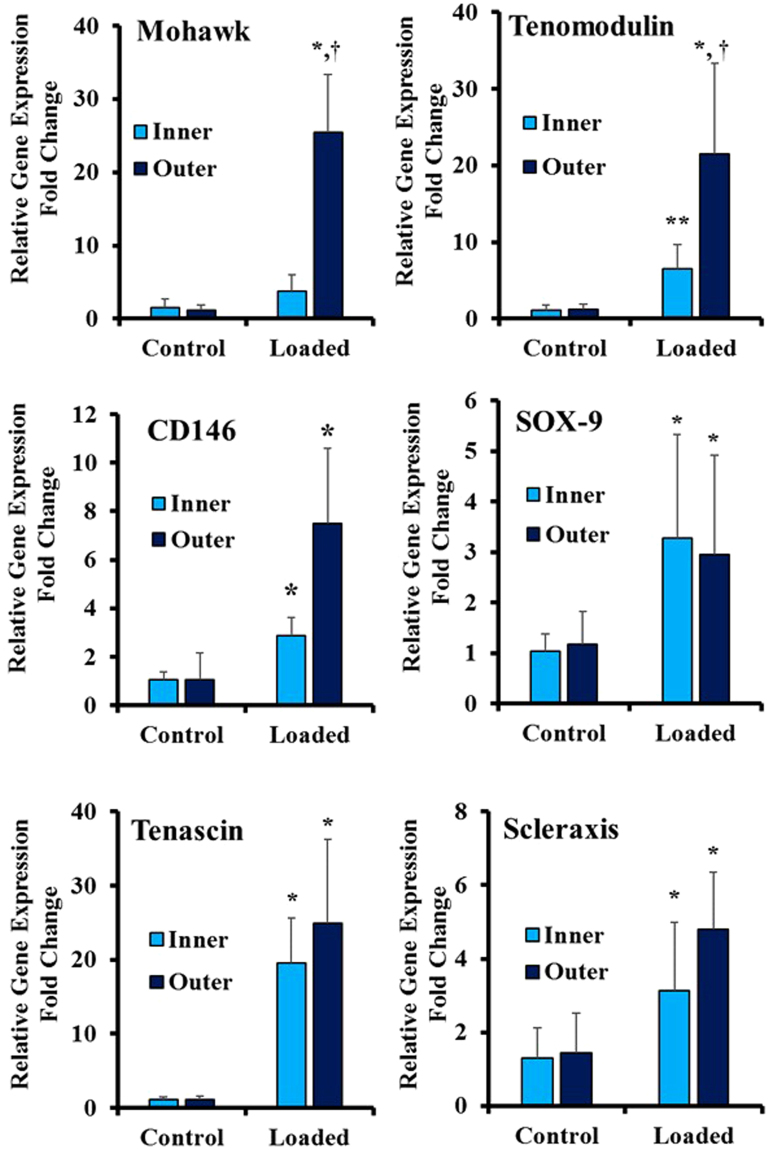
Effect of equiaxial strain on the gene expression profiles of the native AF tissue markers. Loaded samples exhibited higher gene expression compared to control group. Data represent the mean fold change (n = 8), and the error bars represents the standard deviation. *Represents statistical difference from the control (unstrained group) with 0.05 P-value. ^†^Represented a significant difference between the inner and outer regions with a 0.05 P-value.

Also, we investigated the region-specific gene expression associated with inner and outer regions of the native AF tissue for loaded and control samples. Figure 8 shows that Mohawk transcription factor that was recently shown to regulate the maintenance and regeneration of the outer annulus fibrosus tissue^8^ was upregulated in the outer region of the biphasic scaffold, but was not statistically different from control in Collagen inner portion. Similarly, Tenomodulin gene was significantly higher in the outer region compared to the inner region. Both the inner and outer regions were significantly higher than the control group. A recently identified surface marker for AF tissue (CD146) gene expression was also investigated. The results show that CD146 gene expression was up-regulated in the inner and outer regions of the scaffold due to the applied equiaxial strain. As for the chondrogenic marker SOX-9, we observed almost higher gene expression in both the inner and outer regions of the scaffolds. There was no statistical difference between the two loaded regions. Furthermore, we found significantly higher Tenascin gene expression for both the Collagen and PNCOL portions of the scaffold. As for Scleraxis, the gene expression was higher than control for both inner and outer regions.

Our results indicate that the biphasic collagen-PNCOL scaffold possesses a great potential for degenerated AF tissue repair as it maintained the cells under dynamic mechanical environment without causing any adverse effects. Also, we showed significant higher gene expression of the AF tissue markers and higher ECM proteins secretion compared to control group that was cultured under no-loading conditions. Additionally, the biphasic scaffolds possessed aligned fibers and elongated cells morphology which mimics the native environment of the AF tissue. Importantly, we showed that region-specific differentiation that is associated with the inner and outer regions of the native AF tissue could be achieved in the biphasic scaffolds upon applying the equiaxial mechanical strain.

## Discussion

We report for the first time a systematic process to optimize the mechanical loading modality of 3D adipose-derived mesenchymal stem cells (ASCs)-encapsulated collagen and collagen-PNCOL biphasic scaffolds with the goal of introducing a potential repair strategy for the annular defects of the intervertebral disc. Directing the lineage commitment of ASCs can be controlled by soluble factors that may be supplemented to cell culture media or incorporated into tissue-engineered constructs^18,42,43^. However, several studies showed that mechanical cues could also direct the ASCs differentiation potential to a specific lineage. In fact, a recent study reported that mechanical signaling is required to activate the growth factors that maintain the homeostasis of the IVD^44^. However, the nature of mechanical cue is very specific to the tissue of interest. Previous studies showed that moderate mechanical forces are essential for the maintenance of IVD homeostasis. Also, dynamic physiological loading can repair the ECM depletion, which is mostly found in the early stages of IVD degeneration^45–47^. Our literature review revealed that even though there are studies linking uniaxial and biaxial strains to ASCs differentiation^24,31^, there are almost no studies that investigated the effect of equiaxial strain on the differentiation potential of these cells in 3D culture conditions. Closing this gap can introduce new strategies to regenerate AF tissues that undergo equiaxial strain in their native environment.

Our prior *in vitro* ^32^ and *in silico* ^33^ studies showed that the custom-designed equiaxial mechanical loading bioreactor, EQUicycler, could apply homogenous equiaxial strain to various cell types of musculoskeletal origins. The design detail about EQUicycler is provided in Supplementary data (Figures S1 and S2). In the current study, we utilized EQUicycler to apply various equiaxial strains within the physiological mechanical range of native AF tissue^27,28,34–36^. We first identified the mechanical loading regime that can differentiate ASCs within the collagen matrix into AF lineage. We applied 3%, 6%, and 12% equiaxial strain at 0.1 Hz and 1 Hz frequency to the collagen ASCs-encapsulated scaffolds and studied the changes in gene expression and scaffold morphology upon mechanical loading. The gene expression data (Figs 1 and 2) along with secreted protein data (Fig. 3) demonstrated that 6% equiaxial mechanical strain with 1 Hz frequency differentiated ASCs into AF lineage. Once the optimal mechanical loading regime was defined, we applied this loading to the biphasic scaffold (collagen-PNCOL) with two distinctive regions representing outer and inner regions of the native AF tissue.

This comprehensive study demonstrated that mechanical equiaxial strain up-regulated various ECM markers including Aggrecan, Collagen Type-I, II, III, and V and AF tissue markers including Scleraxis, Tenascin, Tenomodulin, Mohawk, and SOX-9 (Fig. 1). We further identified the mechanical strain values that did not upregulate the osteogenic markers to exclude mechanical strains that promote cross-differentiation of bone and AF tissue lineage. The 3% and 6% mechanical strains at 1 Hz frequency did not upregulate the osteogenic markers compared to the other mechanical strains (Fig. 2A). The gene expression data for two catabolic markers (MMP-2 and MMP-13) that are associated with AF degeneration demonstrated down-regulation for 3% strains at 0.1 and 1 Hz, 6%, and 12% strains at 1 Hz. However, MMP-2 expression was upregulated for the 12% and 6% mechanical strains at 0.1 Hz. TIMP-1 expression was upregulated for the 1 Hz mechanically loaded groups. Sowa *et al*.^28^ demonstrated that AF cells express anabolic or catabolic markers depending on magnitude, frequency, and duration of mechanical strain. They demonstrated that low magnitudes and frequencies resulted in catabolic effects. However, they mentioned that their study did not account the effect of matrix-cell interactions since the cells were loaded using a 2D monolayer system. We further investigated the amounts of secreted ECM proteins that were soluble in the constructs culture media to examine if the higher gene expression could translate to protein secretion. Our results that were presented in Fig. 3 demonstrated that loaded collagen constructs had higher normalized collagen as well as sGAG in the culture media compared to the control group.

In addition to gene expression and secreted ECM proteins, we examined the degree of the matrix organization and the morphology of elongated cells within the ASCs-encapsulated collagen scaffolds. Mechanically loaded scaffolds demonstrated highly aligned collagen fibers and elongated cells compared to the control group with random collagen fiber and round cell morphology. The matrix alignment upon mechanical loading can be attributed to a synergetic effect of equiaxial mechanical loading and cell-mediated compaction of collagen fibers, as demonstrated in previous studies^32,48–51^. In the light of gene expression data (Figs 1 and 2) and the soluble ECM proteins (Fig. 3), 6% mechanical strain at 1 Hz was chosen for mechanical loading of the biphasic AF-like tissue scaffold.

**Figure 9:**
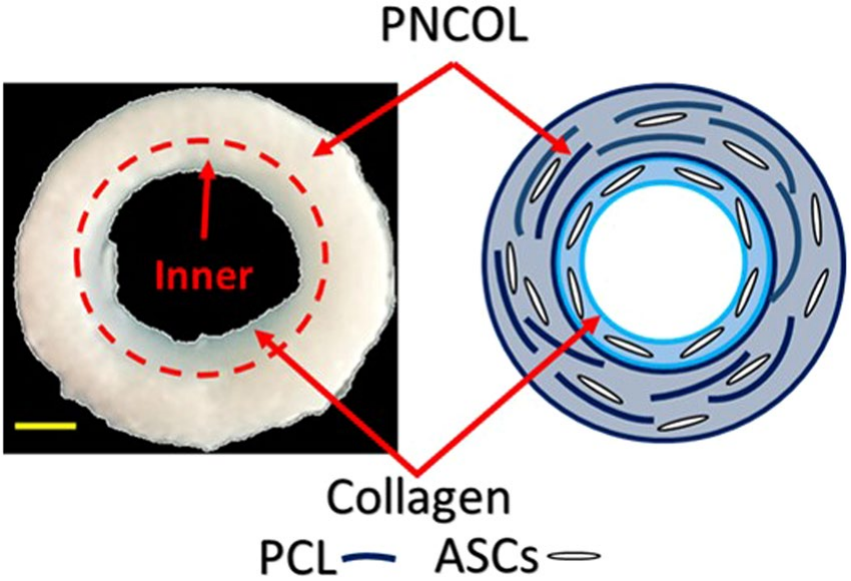
Representative visualization photograph of the biphasic scaffolds after 7 days of mechanical loading in culture conditions and a schematic view of the biphasic scaffolds. Collagen (inner)-PNCOL (outer) layers of the scaffold are depicted. The scale bar represents 4 mm.

The biphasic scaffold is composed of two concentric regions (Fig. 9). The inner region of the scaffold is an ASCs-encapsulated collagen, and the outer region is a nanofibrous matrix called PNCOL composed of polycaprolactone (PCL) nanofibers interspersed within the ASCs-encapsulated collagen matrix. The rationale for using the composite nanofibrous material for biphasic scaffold was to mimic the native AF tissue. AF tissue is a fibrous tissue with softer cartilage-like inner layers and stiffer and ligament-like outer layers^8,52^.

We have applied 6% mechanical strain with 1 Hz frequency and studied the changes in ECM and AF markers for the inner and outer region of the biphasic scaffold. The Mohawk, a newly discovered transcription factor in the outer part of AF tissue, and Tenomodulin, a marker of AF lineage^37^, expressed significantly in the outer region of biphasic scaffold compared to inner region and unstrained biphasic scaffold. This result agrees with the finding of Nakamichi *et al*.^8^ who reported that Mohawk regulates the regeneration of the outer annulus fibrosus of intervertebral discs. Other markers of the AF tissue, SOX-9, Tenascin, Scleraxis, and CD146^8,52–55^, were up-regulated both in the inner and outer regions of the scaffold. The expression of large proteoglycan gene (Aggrecan) increased significantly in the inner region of the biphasic scaffold (Fig. 7). This result can be explained due to the nature of the equiaxial loading imposed by EQUicycler that encounters compressive radial strain gradient from inner to outer regions. The radial strain gradient has maximum absolute value at the inner part and zero value at the free outer region. This mechanical environment mimics the AF tissue in which the NP pressurization applies compressive radial strain and tensile circumferential equiaxial strain on the AF tissue. The radial compressive strain gradient could be responsible for the higher aggrecan expression data in the inner region of the scaffold. The ECM markers besides Aggrecan were upregulated in both inner and outer regions of the biphasic scaffolds compared to control. Yet, there was no statistical difference in their expression for the inner and outer region.

The gene expression results were further checked for protein secretion in the cell culture media. We found a significant increase in the amounts of soluble collagens and sGAG and culture media of loaded biphasic scaffolds compared to control group (Fig. 5). Since the biphasic scaffolds constituted of mainly collagen, we were not able to distinguish the de novo secreted collagen in the scaffolds histological sections which implies a limitation in the current data. The secreted sGAG; however, could be distinguished in the histological sections in which we observed higher amounts of sGAG in the loaded samples compared to control group as seen in Fig. 6. The Mason’s trichrome staining of the biphasic scaffold showed elongated cells and aligned collagen fibers in both the inner and outer regions. However, we observed less degree of alignment in the outer region due to the dispersed nanofibers presence within the collagen. These outcomes can also be augmented by conjugating growth factors to the outer region (PNCOL) of the scaffold as our group reported earlier^56^ to harness the synergetic effect of mechanical and biochemical stimulation. Among the well-studied growth factors that can aid in directing the differentiation of stem cells to regenerate AF is the TGF-β family^44,57,58^; however, equiaxial loading strain that is characteristic of AF native environment can further increase the specificity of the differentiation. Several studies reported that mechanical stimulation of stem cells differentiation acts through TGF-β pathway^44,59,60^. These findings implied that incorporating growth factors such as TGF is not sufficient and that the appropriate mechanical cues are required for a successful IVD regeneration strategy.

As for the limitations of the current study, we did not observe higher proliferation rate for the loaded samples compared to control possibly due to the onset of differentiation^61^. Also, we did not study the mechanisms of gene expression up-regulation suppression that we observed due to applying equiaxial strain. Also, the current study did not distinguish between the different types of secreted collagen at the protein level. Further, we were not able to investigate the amount of secreted de novo collagen within the scaffolds. The study can be extended by incorporating growth factors in the PNCOL portion of the biphasic scaffolds to study the synergetic effects of biochemical and mechanical elicitation of ASCs differentiation to AF-lineage.

## Conclusion

Applying varying magnitudes of equiaxial strain at different frequencies was utilized to investigate the potential differentiation of ASCs to AF phenotype. Based on the gene expression profiles, protein secretions, matrix organization and cells morphology, we choose 6% strain and 1 Hz as the optimum loading modality to induce AF-like cells and matrix. With the goal of introducing a framework strategy to repair the Annulus Fibrosus of the IVD, we utilized the optimized loading modality to a biphasic ASCs encapsulated collagen-PNCOL scaffolds. The results showed region-specific differentiation of ASCs towards the inner and outer regions associated with the AF tissue, higher ECM markers secretion, elongated cells, and aligned matrix organization.

## Methods

### Preparing Adipose-derived Mesenchymal Stem Cells-embedded Collagen Constructs

Adipose-derived mesenchymal stem cells (ThermoFisher, USA) were cultured in MesenPRO RS™ basal media supplemented with MesenPRO RS™ growth supplements (ThermoFisher, USA), 200 mM Glutamine (Sigma-Aldrich, USA) and 1% Penicillin-Streptomycin (ThermoFisher, USA). ASCs-encapsulated collagen scaffolds were prepared by embedding passage 4 ASCs at 10^6^ cells/ml seeding density in 3 mg/ml collagen Type-I solution. The cell-encapsulated collagen solutions were then deposited around the silicone posts, polymerized at 37oC for 1 hour and incubated in the culture media.

### Preparing of Adipose-derived Mesenchymal Stem Cells-embedded biphasic Collagen-PNCOL Constructs

Polycaprolactone (PCL, Mw = 45,000, Sigma-Aldrich, USA) nanofibers were fabricated by electrospinning as we should before^56,62,63^. The concentration of PCL (3% w/v) was chosen based on our previous studies that optimized the PCL w/v % to maximize the mechanical properties of the scaffold, preserve the cellular functions and the bioactivity of loaded growth factors. Collagen ASCs-encapsulated constructs were prepared and deposited around silicone posts as previously described. Polymerized constructs were incubated for two days in cell culture conditions. PNCOL ASCs-encapsulated constructs were prepared and deposited around polymerized collagen rings to form a one unit scaffold composed of two concentric layers. Biphasic scaffolds were then incubated overnight before they were subjected to mechanical loading. Figure 9 depicts a visualization of the biphasic scaffolds collected after 7 days of mechanical loading in culture conditions and a schematic representation of the biphasic scaffolds.

### Mechanical Loading of ASCs-encapsulated constructs

Collagen ASCs-encapsulated constructs were subjected to 3%, 6%, or 12% equiaxial strain at either 0.1 Hz or 1 Hz loading frequency. The native annulus fibrosus physiology was taken as the reference to decide the mechanical loading modalities that would be applied on the scaffolds. For the biphasic collagen-PNCOL scaffolds, the samples were subjected to 6% strain at 1 Hz loading frequency. The loading was applied using EQUicycler (EQUicycler design is presented in the supplementary material) for 2 hours/day for a period of 7 days. After 7 days of loading, the samples were incubated for 24 hours and then harvested for characterization assays including cell proliferation, matrix organization, soluble secreted GAGs, soluble secreted Collagens, and gene expression profiles.

### Assessing the relative gene expression response to equiaxial strain using

The differentiation response of ASCs encapsulated in collagen or PNCOL scaffolds due to the application of different mechanical stimulation modalities was investigated by performing gene expression fold change analysis of several genes using quantitative real-time polymerase chain reaction (RT-qPCR). First, the scaffolds were mechanically disrupted, then the RNA was extracted using TRIzol reagent (ThermoFisher, USA). The isolated RNA was reverse transcribed to cDNA using Omniscript RT kit (Qiagen, USA) per the manufacturer’s protocols. RT-qPCR was performed using SYBR Green PCR master mix (ThermoFisher, USA) in the iCycler iQ detection system (Bio-Rad, USA). The relative gene expression for fold difference between strained samples and non-loaded control samples was obtained using the ΔΔC_t_ method. In the ΔΔC_t_ method, Glyceraldehyde-3-phosphate dehydrogenase (GAPDH) was used as the housekeeping normalizing gene. The primers’ sequences for each gene were obtained from literature^64–70^ and synthesized by IDT (USA). The primers sequences are given in the supplementary material Table S1.

### Assessing the secreted ECM proteins within ASC-encapsulated 3D collagen and Collagen-PNCOL scaffolds culture media

To investigate the effect of mechanical loading on the secreted ECM proteins, Dimethylmethylene Blue Assay (DMMB) assay and Sircol assay (Biocolor, UK) were used to quantify the soluble sGAG and collagens, respectively, in the cell culture media. At the day of characterization, the cell culture media were collected. A portion of the media was mixed with either DMMB dye or Sircol dye and incubated with moderate agitation at room temperature for 30 min. Upon incubation, the solution was centrifuged to form a pellet of sGAG or collagens that bound to the respective dye. The pellet was washed in ice-cold acid-salt solution, centrifuged and resuspended in 10% SDS for DMMB assay or Alkali solution for Sircol assay. For DMMB assay, absorbance at 656 wavelength while for Sircol assay absorbance at 555 wavelength was quantified using a microplate reader (SOFTmax Pro, USA). Absorbance was converted to sGAG concentration or Collagen concentration using a calibration curve obtained using different concentrations of chondroitin sulfate (Sigma-Aldrich, USA) or Type-I collagen (Corning, USA), respectively. The sGAG or collagen concentrations were normalized using the total protein in the media quantified using UV 280 wavelength absorbance and converted to protein concentration through a calibration curve obtained by diluting a stock solution of Bovine Serum Albumin (BSA, Sigma-Aldrich, USA).

### Assessing the Matrix organization within ASC-encapsulated 3D collagen and Collagen-PNCOL scaffolds

The matrix organization and secreted sGAG within the ASC-encapsulated collagen scaffolds under various mechanical loading regimes was visualized using histological analyses and Mason’s Trichrome or Alcian Blue staining as before^32^.

### Statistical analysis

Eight samples (n = 8) were used for all assays. Statistical analysis was conducted by ANOVA followed by Fisher LSD post-hoc using SPSS Statistics software (IBM, USA). The data is reported as the mean ± standard deviation. * indicated significant difference with respect to 0% strain group (control) with P < 0.05. § represented significant difference between 0.1 Hz and 1 Hz (at the same strain magnitude), † represented significant difference between 3% and 6% strain groups, ‡ represented significant difference between 3% and 12% strain groups while # represented significant difference between 6% and 12% strain groups (at the same frequency), each with a 0.05 P-value. For the biphasic scaffolds, † represented a significant difference between the inner and outer layers with a 0.05 P-value.

## Electronic supplementary material

Supplementary Data
