# Supplementary material for: Equiaxial Strain Modulates Adipose-derived Stem Cell Differentiation within 3D Biphasic Scaffolds towards Annulus Fibrosus
Source: Sci Rep. 2017 Oct 9;7:12868. doi: 10.1038/s41598-017-13240-3 (PMC5634474; doi:10.1038/s41598-017-13240-3)
Supplement: Supplementary file 1 — Supplementary Data [file 41598_2017_13240_MOESM1_ESM.doc]

**Equiaxial Strain Modulates Adipose-derived Stem Cell Differentiation within 3D Biphasic Scaffolds towards Annulus Fibrosus**

Mostafa Elsaadany^1^, Kayla Winters^1^, Sarah Adams^1^, Alexander Stasuk^1^, Halim Ayan^1^, Eda Yildirim-Ayan^1*^

^1^Department of Bioengineering, University of Toledo, Toledo, OH, USA

^*^Corresponding author: [Eda.yildirimayan@utoledo.edu](mailto:Eda-yildirimayan@utoledo.edu)

**Supplementary Material**

**S1.1 Loading platform: EQUicycler**

A modified version of the equiaxial strain loading platform that we presented earlier [1, 2] is utilized in the current study to provide more flexibility in the selection of the applied mechanical strain and its frequency. A more robust driving mechanism that employs a two-phase high torque stepper motor and drive screw was utilized. The stepper motor is controlled by a LabVIEW-based software provided by the manufacturer (Lin Engineering, USA). The driving mechanism allows for a continuous range of the applied strain rather having to machine a new cam that corresponds to a certain strain. Additionally, a new polymerization chamber that employs a mechanical sealing system during the polymerization of the cell-encapsulated constructs. The new sealing system eliminates the use of chemical sealants that requires tedious cleaning process and increases the risk of contamination.

A schematic view of the polymerization apparatus of the new design is depicted in Figure S 1, and the whole working design assembly is shown in Figure S 1. All the system components were designed and visualized using SolidWorks 3D modeling package (SolidWorks, MA). Shop drawings were generated using Solidworks, and individual components were manufactured and assembled at a high-precision local machine shop (Alton Precision Machining, USA). The parts of the bioreactor are made of polycarbonate, or stainless steel (McMaster-Carr, IL). As seen on Figure S 1, The culture plates (1) are attached to a polymerization mold (2) that has ten annular holes concentric with the silicone posts (3). The assembly is secured via five Philips screws that press the base of the silicone posts and prevent leakage of the cell-encapsulated constructs during polymerization. After polymerization, the molds are removed and the culture plate and placed on EQUicycler. As seen in Figure S 2, the new design utilized a precise coupled stepper motor-screw mechanism that is attached to the pushing plate. The pushing plate moves upwards when the motor rotates clockwise and reverse the motion vice versa. While the pushing plate moves upwards, the culture plate moves along and the silicone posts are compressed against a fixed plate. A precise displacement that corresponds to a certain strain is calculated using finite element analysis and inputted as a number of steps to the stepper motor. The motion frequency can be controlled using the LabVIEW interface of the motor controller.


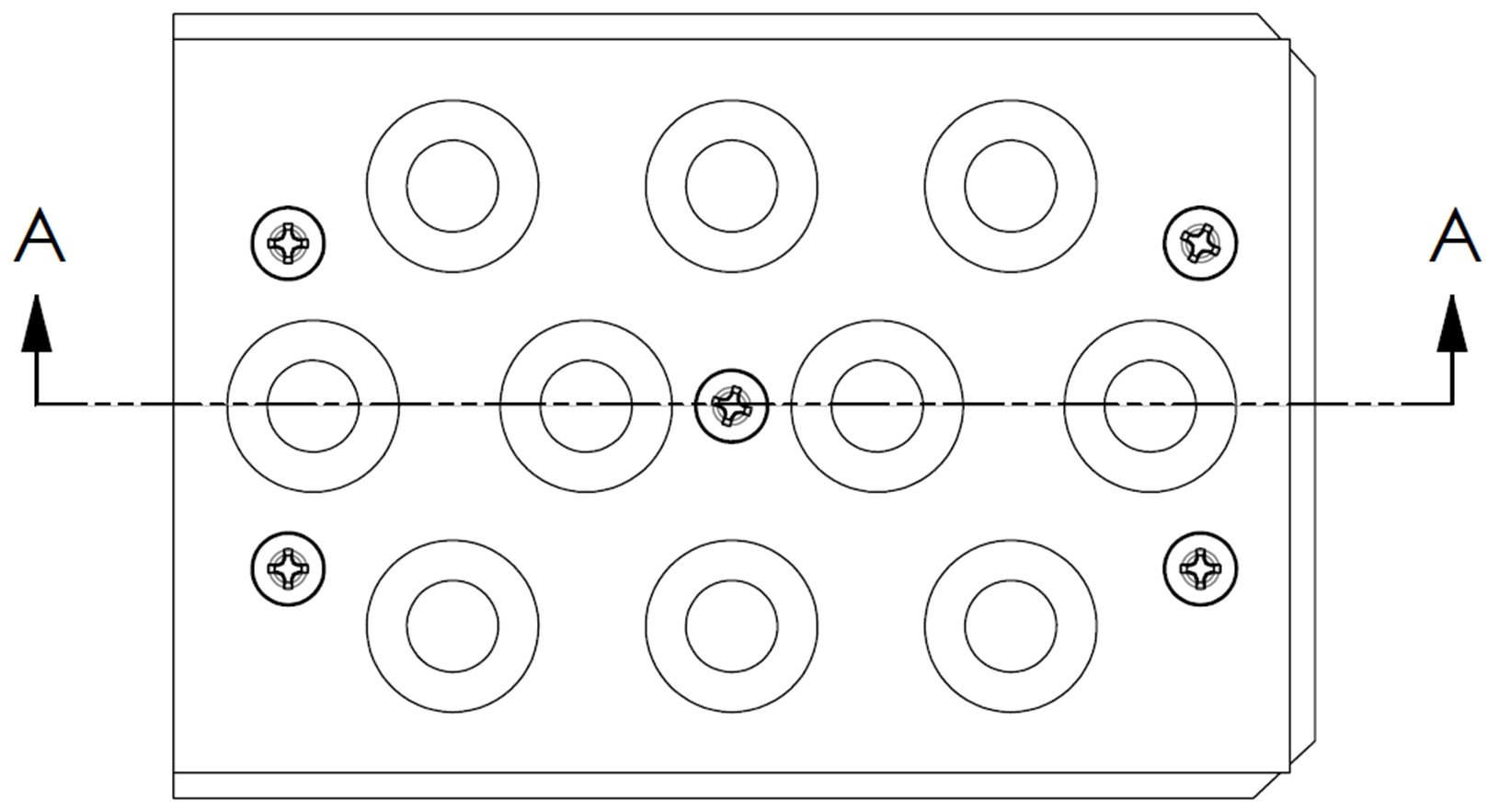


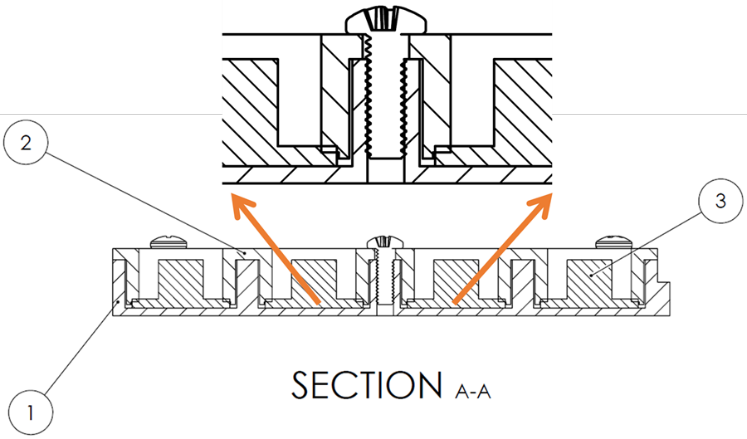


**Figure S 1.** Top view (on top) and sectional front view of the polymerization chamber (bottom) of EQUicycler.


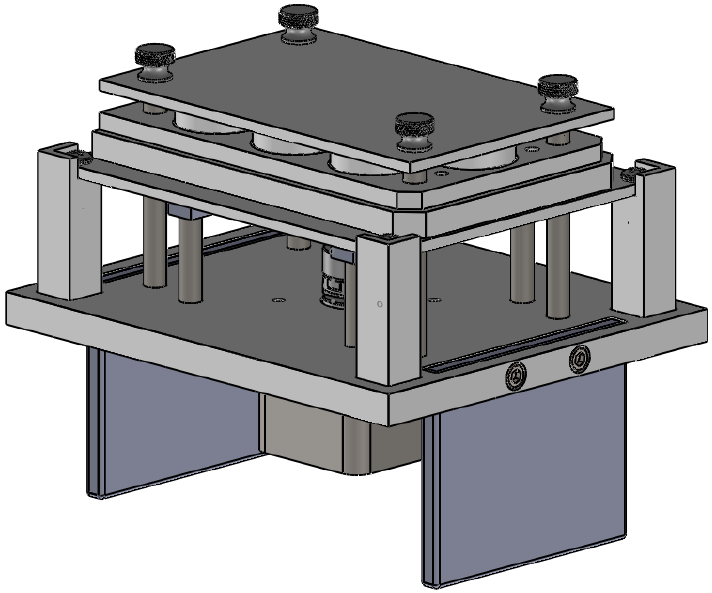

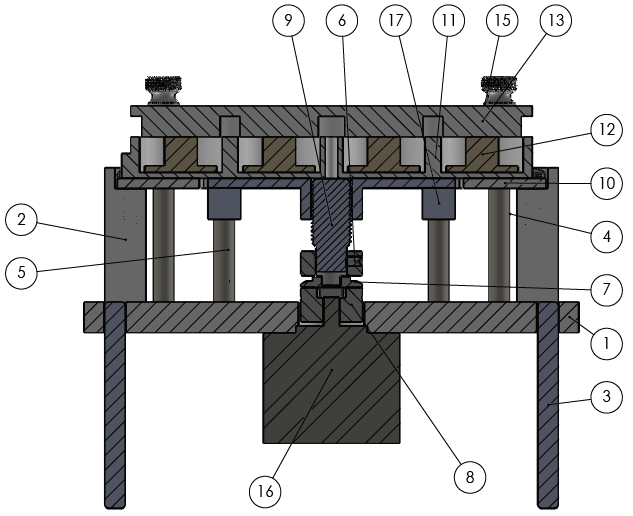


**Figure S 2.** Schematic isometric view of EQUicycler second design. Sectional front view of the assembly (1) Base, (2) Support Columns for the culture plates (3) Supports (4) Guiding rods for culture plates, (5) Guiding rods for the pushing plate, (6, 7, 8) coupling system, (9) Driving Screw (10) supporting plate, (11) culture plate (12) Silicone posts, (13) Fixed Plate, (15) Thump Screws, (16) Stepper Motor, and (17) Pushing Plate.

**S1.2 Effect of equiaxial mechanical loading on ACSs proliferation within biphasic scaffold**

Adipose-derived Mesenchymal stem cells proliferation within the strained and control scaffolds (0% strain) was estimated by quantifying the amount of DNA within the scaffolds. DNA count was obtained using PicoGreen dsDNA kit (ThermoFisher, USA). First, the cells were liberated from the collagen scaffolds by mechanical disruption and then resuspended in lysis buffer (200 µg/ml of proteinase-K, 50 mM Tris-HCl, 1 mM CaCl_2_, pH=8). The cell lysate was then incubated at 55 °C overnight. Dilution of the cell lysate samples (1:10 in TE buffer) was incubated with 1:1 ratio of 1:200 dilution of PicoGreen dye for five minutes at room temperature. After the incubation period, fluorescence at 480/520nm excitation/emission wavelengths was quantified using a microplate fluorometer (Wallac 1420, USA). Fluorescence intensity was converted into cell number using a calibration curve obtained by diluting a stock solution of a certain cell density and obtaining their corresponding DNA count to compare with the initial seeding density.

To investigate the ASCs proliferation within the inner (Collagen) and outer (PNCOL) layers of the biphasic scaffolds, DNA counts were obtained using PicoGreen assay and correlated to cell number as previously described. Error: Reference source not found presents the cell counts of both the control (unstrained) and loaded samples (6%, 1 Hz).


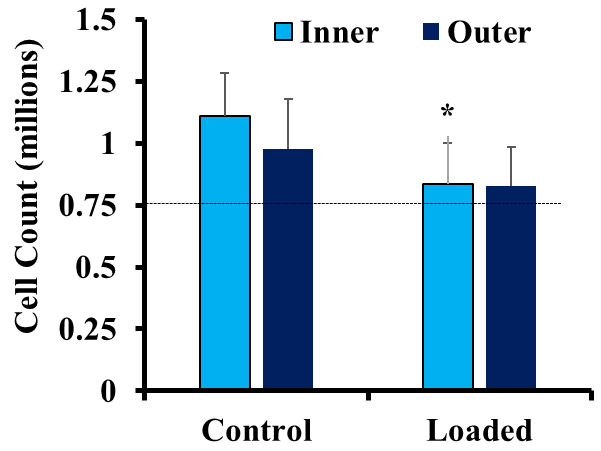


**Figure S 3. Effect of equiaxial loading on ASCs proliferation within 3D biphasic Collagen-PNCOL scaffolds.** Loaded samples show limited proliferation than control. No statistical difference was found between the inner and outer parts of the scaffolds. * represents the statistical difference between the collagen part of the biphasic control scaffolds.

The loaded samples showed limited proliferation compared to control group. For the inner portion of the biphasic scaffolds (collagen section), the cell number was significantly lower than the control while for the outer part of the biphasic scaffold (PNCOL section), the cell count was not statistically different from the control. We did not observe higher proliferation rates for the loaded samples compared to control in contrast with our previous study [2] that showed higher proliferation rate of various musculoskeletal cell lines loaded under equiaxial strain. This discrepancy can be attributed to the fact that, in the previous study, we used immortalized terminally differentiated cell lines that had unlimited proliferation capacity compared to ASCs that were utilized in the current work. Also, there might be an effect of the cells onset of differentiation that might slow down their proliferation rate [3]. Additionally, a recent study of Egea et al. [4] showed that TIMP-1 suppresses the proliferation of mesenchymal stem cells. Our gene expression data in Error: Reference source not found showed that TIMP-1 was upregulated which might explain why we had slower proliferation in the loaded samples.

**Table S1** Forward and reverse primers used for RT-qPCR.

| **Gene** | **Forward Primer** | **Reverse Primer** | **Ref** |
| --- | --- | --- | --- |
| GAPDH | 5’ AGAAGGCTGGGGCTGATTTG 3’ | 5’ AGGGCCCATCCACAGTCTTC 3’ | [5] |
| COL-I | 5’ GGCTCCTGCTCCTCTTAGCG 3’ | 5’ CATGGTACCTGAGGCCGTTC 3’ | [6] |
| COL-II | 5’ GGCAATAGCAGGTTCACGTACA 3’ | 5’ CGATAACAGTCTTGCCCCACTT 3’ | [7] |
| COL-III | 5’ CAGCGGTTCTCCAGGCAAGG 3’ | 5’ CTCCAGTGATCCCAGCAATCC 3’ | [6] |
| COL-V | 5’ GACTGTGACACCGCAGTACC 3’ | 5' GTCTTCGTAGTAGGGGTATTCGTAG 3’ | [8] |
| BGN | 5' GGAACCCACTGGAGAACAGT 3’ | 5' AGGGTCTCAGGGAGGTCTTT 3’ | [9] |
| ACAN | 5’ CACTGTTACCGCCACTTCCC 3’ | 5’ ACCAGCGGAAGTCCCCTTCG 3’ | [10] |
| TCN | 5’ GGTGGATGGATTGTGTTCCTGAGA 3’ | 5’ CTGTGTCCTTGTCAAAGGTGGAGA 3’ | [6] |
| SCX | 5’ ACACCCAGCCCAAACAGA 3’ | 5’ GCGGTCCTTGCTCAACTTTC 3’ | [6] |
| TNMD | 5’ CCATGCTGGATGAGAGAGGT 3’ | 5’ CTCGTCCTCCTTGGTAGCAG 3’ | [10] |
| MKX | 5' TCAAGGACAACCTCGGCCTG3' | 5'-ACGGGTTGTCACGGTGCTTG-3 | [8] |
| CD146 | 5’ ACCCTGAATGTCCTCGTGAC 3’ | 5’ TCTCTGTGGAGGTGCTGTTG 3’ | [11] |
| Sox9 | 5’ CACACAGCTCACTCGACCTTG 3’ | 5’ TTCGGTTATTTTTAGGATCATCTCG 3’ | [7] |
| RUNX2 | 5’ CAACCACAGAACCACAAGTGC 3’ | 5’ TGTTTGATGCCATAGTCCCTCC 3’ | [6] |
| ALP | 5’ GATCTTCTTTCTCCTTTGCCTGG 3’ | 5’ TGTTTGCAGTGGTGGTTCTGGCA 3’ | [12] |
| MMP-2 | 5’ ACAATGAGGTGAAGAAAATGGA 3’ | 5’ AGGTAATAGGCACCCTTGAAGAAGTA 3’ | [13] |
| MMP-13 | 5’ ACTGAGAGGCTCCGAGAAATG 3’ | 5’ GAACCCCGCATCTTGGCTT 3’ | [6] |
| TIMP-1 | 5’ AGTCAACCACGACCACCTTATACCA 3’ | 5’ TTTCAGAGCCTTGGAGGAGCTGGTC 3’ | [14] |

**References in the Supplementary Material**

[1] M. Elsaadany, K.C. Yan, E. Yildirim-Ayan, Predicting cell viability within tissue scaffolds under equiaxial strain: multi-scale finite element model of collagen–cardiomyocytes constructs, Biomech Model Mechan (2017) 1-15.

[2] M. Elsaadany, M. Harris, E. Yildirim-Ayan, Design and validation of equiaxial mechanical strain platform, EQUicycler, for 3D Tissue Engineered Constructs, Biomed Res Int 2017 (2017).

[3] M. Elsaadany, G. Subramanian, H. Ayan, E. Yildirim-Ayan, Exogenous nitric oxide (NO) generated by NO-plasma treatment modulates osteoprogenitor cells early differentiation, Journal of Physics D: Applied Physics 48(34) (2015) 345401.

[4] V. Egea, S. Zahler, N. Rieth, P. Neth, T. Popp, K. Kehe, M. Jochum, C. Ries, Tissue inhibitor of metalloproteinase-1 (TIMP-1) regulates mesenchymal stem cells through let-7f microRNA and Wnt/β-catenin signaling, Proceedings of the National Academy of Sciences 109(6) (2012) E309-E316.

[5] L. Guo, B. Xie, Z. Mao, Autophagy in premature senescent cells is activated via AMPK pathway, Int J Mol Sci 13(3) (2012) 3563-3582.

[6] G. Yang, B.B. Rothrauff, H. Lin, R. Gottardi, P.G. Alexander, R.S. Tuan, Enhancement of tenogenic differentiation of human adipose stem cells by tendon-derived extracellular matrix, Biomaterials 34(37) (2013) 9295-9306.

[7] N. Indrawattana, G. Chen, M. Tadokoro, L.H. Shann, H. Ohgushi, T. Tateishi, J. Tanaka, A. Bunyaratvej, Growth factor combination for chondrogenic induction from human mesenchymal stem cell, Biochemical and biophysical research communications 320(3) (2004) 914-919.

[8] R. Nakamichi, Y. Ito, M. Inui, N. Onizuka, T. Kayama, K. Kataoka, H. Suzuki, M. Mori, M. Inagawa, S. Ichinose, Mohawk promotes the maintenance and regeneration of the outer annulus fibrosus of intervertebral discs, Nature Communications 7 (2016).

[9] M. Liu, J. Dai, Y. Lin, L. Yang, H. Dong, Y. Li, Y. Ding, Y. Duan, Effect of the cyclic stretch on the expression of osteogenesis genes in human periodontal ligament cells, Gene 491(2) (2012) 187-193.

[10] M.F. Pietschmann, M.U. Wagenhäuser, M.F. Gülecyüz, A. Ficklscherer, V. Jansson, P.E. Müller, The long head of the biceps tendon is a suitable cell source for tendon tissue regeneration, Archives of medical science : AMS 10 (2014) 587-96.

[11] S. Hörl, A. Ejaz, S. Ernst, M. Mattesich, A. Kaiser, B. Jenewein, M.E. Zwierzina, S. Hammerle, C. Miggitsch, M.C. Mitterberger-Vogt, C. Krautgasser, G. Pierer, W. Zwerschke, CD146 (MCAM) in human cs-DLK1−/cs-CD34+ adipose stromal/progenitor cells, Stem Cell Research 22 (2017) 1-12.

[12] J.W. Park, J.Y. Suh, H.J. Chung, Effects of calcium ion incorporation on osteoblast gene expression in MC3T3‐E1 cells cultured on microstructured titanium surfaces, J Biomed Mater Res A 86(1) (2008) 117-126.

[13] H. Alfredson, M. Lorentzon, S. Bäckman, A. Bäckman, U.H. Lerner, cDNA‐arrays and real‐time quantitative PCR techniques in the investigation of chronic Achilles tendinosis, J Orthop Res 21(6) (2003) 970-975.

[14] T. Kubota, Y. Matsuki, T. Nomura, K. Hara, In situ hybridization study on tissue inhibitors of metalloproteinases (TIMPs) mRNA‐expressing cells in human inflamed gingival tissue, J Periodontal Res 32(5) (1997) 467-472.
